# Identification of Pentatricopeptide Repeat Proteins in the Model Organism *Dictyostelium discoideum*

**DOI:** 10.1155/2013/586498

**Published:** 2013-08-12

**Authors:** Sam Manna, Jessica Brewster, Christian Barth

**Affiliations:** Department of Microbiology, Thomas Cherry Building, La Trobe University, Kingsbury Drive, Bundoora, VIC 3086, Australia

## Abstract

Pentatricopeptide repeat (PPR) proteins are RNA binding proteins with functions in organelle RNA metabolism. They are found in all eukaryotes but have been most extensively studied in plants. We report on the identification of 12 PPR-encoding genes in the genome of the protist *Dictyostelium discoideum*, with potential homologs in other members of the same lineage and some predicted novel functions for the encoded gene products in protists. For one of the gene products, we show that it localizes to the mitochondria, and we also demonstrate that antisense inhibition of its expression leads to slower growth, a phenotype associated with mitochondrial dysfunction.

## 1. Introduction

Mitochondria contain their own genome and, as is the case for any other genome, must maintain tight control over the expression of their encoded gene products. Mitochondrial genes typically encode either components of the respiratory chain for ATP synthesis or the mitochondrial translation machinery. Regulating the expression of such genes is therefore essential for normal cell function, as aberrations in the regulation of mitochondrial gene expression can result in disease [1, 2]. Similarly to nuclear and bacterial gene expression, post-transcriptional regulation is one of the most important stages of mitochondrial gene expression. This can include processing of polycistronic transcripts and liberation of structural RNAs, excision of introns, RNA editing, and stability modifications such as polyadenylation [2]. 

Given that these post-transcriptional processes are highly diverse, one would expect such functions to be catalysed by many different proteins. Indeed, each post-transcriptional event often involves several proteins, amongst which a large family of helical repeat proteins have been found to play important roles in organelle gene expression. These rather complex proteins are known as pentatricopeptide repeat (PPR) proteins and were originally identified during the sequencing of the genome of the model plant *Arabidopsis thaliana *[3]. The PPR family is now known as one of the largest protein families to exist in angiosperms with over 450 PPR-encoding genes identified in *A. thaliana *[4]. 

PPR proteins are characterised by a 35 amino acid motif, often repeated in tandem a variable number of times [3, 5]. Each PPR motif consists of two antiparallel *α*-helices, which interact with each other [3, 5]. The series of *α*-helices form a superhelix containing a groove, which can bind its RNA ligand in a sequence-specific manner [5–7]. Most PPR proteins function as molecular adaptors in the recruitment of catalytic enzymes or effector proteins to target transcripts [5, 7]. Two classes of PPR proteins exist. The P class is characterised by the canonical 35 amino acid motif and typically lacks additional domains [5]. The second class, the PLS class, consists of slightly longer and shorter PPR motifs, as well as C-terminal domains such as the E, E+, and DYW domains, which often have prominent roles in RNA editing [5]. Indeed, the presence of PLS class PPR proteins, originally believed to be exclusive to plants, correlates strongly with the occurrence of organelle RNA editing, while these proteins are typically absent in organisms where organelle RNA editing does not occur [8, 9]. Although not as prevalent as in plants, PPR proteins are found in all eukaryotes, where they have specific roles in post-transcriptional regulation of organelle gene expression. Such functions include processing, splicing, RNA editing, stabilisation, polyadenylation, and translational activation [5, 7]. Although several of these functions are regulated by PPR proteins in plants, the most common function for plant PPR proteins seems to be in RNA editing, a process which is rather common in plant organelles [5, 10]. In humans, only seven PPR proteins have been identified. They have been shown to regulate the mitochondrial transcriptome not via RNA editing, but rather through transcription and transcript processing, RNA stability, polyadenylation, and translation [11–15]. 

While the knowledge of PPR protein structure and function in non-plant organisms is expanding exponentially, little is known about the significance of these proteins in the mitochondria of protozoa. In the protists, PPR proteins have been studied mainly in trypanosomatids, where more than 30 PPR genes have been identified, a uniquely high number for a non-plant organism [16–19]. Most of these PPR proteins play roles in either the stabilisation or polyadenylation of kinetoplast transcripts, and they often lack additional C-terminal domains [16–19]. While studies into the heterolobosean protist *Naegleria gruberi *have also identified an unexpectedly high number of PPR-encoding genes, in contrast to trypanosomes a large subset of the gene products belongs to the DYW subclass of the PLS group and has thus been implicated in RNA editing [20, 21]. Despite the identification of PPR genes in *N. gruberi*, none of their gene products have been functionally characterised, and therefore the question remains whether transcript stabilisation and editing are the main functions of PPR proteins in protists.

*Dictyostelium discoideum *is a cellular slime mould belonging to the Amoebozoa and is a widely accepted and well-established model for studying mitochondrial genetics and disease [22, 23]. Transcription of the mitochondrial genome in *D. discoideum *has been studied in detail, and some of the core components mediating the transcription process have been identified. In *D. discoideum *mitochondria, transcription is initiated at a single site and the transcriptome is subjected to several post-transcriptional modifications including processing and intron splicing, as well as a single nucleotide RNA editing event that occurs in the transcript of the mitochondrial *rns* gene [24–28]. However, very little is known about the proteins that regulate these post-transcriptional events, and the existence and potential role of PPR proteins in mitochondrial RNA metabolism have not been investigated in this organism. Here, we describe the identification of genes of the PPR protein family in *D. discoideum*. We found 12 potential PPR proteins encoded in the* D. discoideum *genome, and some of these proteins show significantly different features compared to other known PPR proteins. One of the *D. discoideum *proteins has been characterised in detail, confirming its mitochondrial localisation. We also demonstrate that antisense inhibition of its expression leads to growth defects, a phenotype associated with mitochondrial dysfunction. While the phenotypic changes resulting from antisense inhibition of gene expression of one of these PPR proteins confirm the importance of these proteins in mitochondrial function, their specific role in post-transcriptional regulation of the *D. discoideum *mitochondrial transcriptome still remains to be determined. 

## 2. Materials and Methods

### 2.1. Strains and Culture Conditions

*D. discoideum *strain AX2 and all transformants were grown to a density of 2–5 × 10^6^ cells/mL in HL-5 medium at 21°C [29, 30]. For non-axenic culture, AX2 and all derivatives were grown on SM plates with *Klebsiella aerogenes *lawns [31] unless otherwise stated. 

### 2.2. Transformation of *D. discoideum *with Vector DNA

The calcium phosphate precipitation method was used to transform *D. discoideum *with vector DNA as described previously [32] using 20 *μ*g of vector DNA. Transformants were isolated on *Micrococcus luteus *lawns on SM plates supplemented with 20 *μ*g/mL G-418. [33]. 

### 2.3. Fluorescence Microscopy

To determine the subcellular localisation of PtcB, *D. discoideum *transformants expressing a PtcB:GFP fusion protein were analysed via fluorescence microscopy as described previously [34, 35]. Aliquots of the axenically grown transformant culture (~3 mL) were transferred into a 6-well plate (BD Biosciences) containing coverslips, and the cells were allowed to settle. The medium was removed and the mitochondria were stained with 100 nM MitoTracker (Life Technologies) in Lo-Flo HL-5 medium for 1 hour. Unbound MitoTracker was removed by washing the cells four times with Lo-Flo HL-5 and twice with phosphate buffer. The cells were subsequently fixed by placing the coverslips for 15 minutes upside down onto a 1% agarose gel in phosphate buffer containing 3.7% paraformaldehyde, after which the cells were washed four times with phosphate buffered saline (PBS). Coverslips were rinsed with Milli-Q sdH_2_O and mounted for microscopy with 90% glycerol in PBS. 

### 2.4. Analysis of Growth Rates on Bacterial Lawns

Growth of *D. discoideum *cells was analysed by measuring plaque expansion rates on bacterial lawns as described previously [36]. Briefly, *D. discoideum *cells of interest were collected from the leading edge of a previously grown plaque on *K. aerogenes *lawns. The cells were then used to inoculate normal agar plates with pregrown *Escherichia coli *B2 lawns. The diameter of the plaques was measured every 8 or 16 hours for 7 days to calculate the mean plaque expansion rate (mm/hour) as an estimate of growth. 

### 2.5. Quantitative PCR

The number of vector copies of the *ptcB *antisense construct in each transformant was determined using qPCR. The qPCR reactions were performed using SsoAdvanced SYBR Green Supermix (Bio-Rad). Total gDNA extracted from each antisense transformant and from wild type cells was used as template along with primers specific to the cloned portion of the *ptcB *gene. Cycling conditions were as follows: initial denaturation at 95°C for 10 minutes and then 40 cycles of denaturation at 95°C for 15 seconds, followed by annealing and primer extension at 60°C for 1 minute. All transcript levels were normalised to the single copy number *β*-tubulin (*tubB*) gene. 

## 3. Results and Discussion

### 3.1. Identification of PPR Proteins in *D. discoideum*

**Figure 1:**
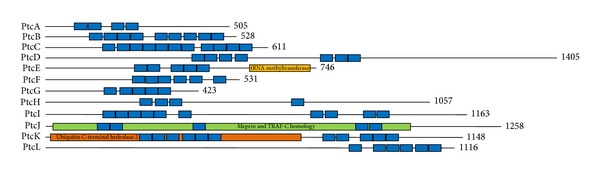
Predicted domain architecture of *D. discoideum *PPR proteins, PtcA-L. Blue boxes represent PPR motifs. The amino acid length of each protein is indicated at the C-terminus of each protein. Also displayed are the putative tRNA methyltransferase (yellow), MATH-like (green), and ubiquitin hydrolase-like (orange) domains of PtcE, PtcJ, and PtcK, respectively.

We analysed the *D. discoideum* genome for any PPR-encoding genes and identified 12 gene sequences coding for putative helical repeat containing proteins. Analysis of the protein sequences using the bioinformatics tool TPRpred [37] confirmed that all candidates contained putative PPR motifs (Table 1). The candidates were named pentatricopeptide repeat containing proteins A-L (PtcA-L). They range in size from 423 to 1405 amino acids, and based on the TPRpred analysis, each contains anywhere from 4 to 11 canonical P class PPR motifs, a typical range for a non-plant PPR protein. The number of PPR proteins identified in *D. discoideum* was also consistent with that observed in other non-plant eukaryotes but was significantly less than the number of PPR proteins observed in other protists such as trypanosomatids and heterolobosea. We did not identify any PLS class-specific features in the PPR protein candidates (Figure 1). The lack of PLS class PPR proteins in *D. discoideum *suggests that PPR proteins are not involved in RNA editing, which correlates well with the rather infrequent occurrence of editing in *D. discoideum *mitochondrial transcripts. This is in contrast to plants and *N. gruberi,* which contain PLS class PPR proteins known to be involved in RNA editing [5, 20, 21]. 

Although most of the identified PPR proteins appear to lack any additional C-terminal domains, one candidate, PtcE, contains a putative C-terminal tRNA m^7^G46 methyltransferase domain. PtcE is, therefore, predicted to catalyse the methylation of mitochondrial tRNA species which contain a guanosine residue at position 46, a role that has not previously been reported for any other PPR protein. 

**Figure 2:**
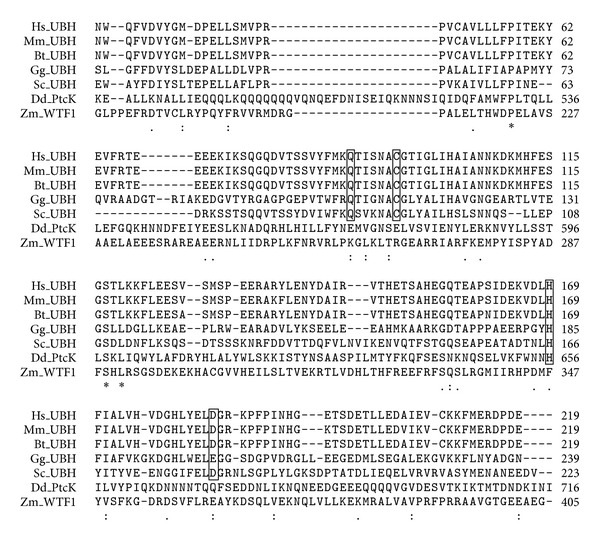
Amino acid sequence alignment of PtcK from *D. discoideum *(Dd) with ubiquitin hydrolases (UBHs) from other organisms. Sequences used in the alignment include UBHs from *Homo sapiens *(Hs, accession number NP_005993), *Mus musculus* (Mm, accession number AAF64193), *Bos Taurus* (Bt, accession number NP_001035631), *Glomerella graminicola* (Gg, accession number EFQ25707), *Saccharomyces cerevisiae* (Sc, accession number EDN63415), and WTF1, a PORR-containing protein from *Zea mays* (Zm, accession number ACI96105). Only the relevant portion of the alignment is shown. Boxed residues indicate conserved amino acids required for ubiquitin hydrolase activity while identical (∗), conserved (:), and semiconserved (.) amino acids are also denoted.

PtcK has a putative ubiquitin carboxyl-terminal hydrolase 2 domain. However, it is noteworthy that PtcK only displays weak similarity to ubiquitin hydrolases and thus may contain a non-functional domain or a similar sequence by chance. Although not homologous, PtcK exhibits similarity to several members of a PPR-like family of plant organelle RNA binding proteins (Figure 2), which contain a plant organelle RNA recognition (PORR) domain (formerly known as domain of unknown function 860 or DUF860). These RNA binding domains are thought to be exclusive to plants, and like the domain in PtcK they not only display weak similarity to ubiquitin hydrolases, but also lack most of the catalytic residues (Figure 2) required for such activity [38, 39]. Additionally, the RNA binding surface of PORR proteins is similar to that of repeated helical motifs, such as PPR motifs, and they have been shown to mediate several aspects of organelle gene expression at the RNA level [38, 39]. Only two members of this family have been characterised and both mediate splicing of introns in organelle transcripts [38, 40]. Although PtcK may not be a member of this family, the features it has in common with the PORR family in addition to the presence of PPR motifs not only imply a similar function for PtcK in mitochondrial gene expression, but also demonstrate a potential evolutionary link between PPR proteins and the PORR family. In fact, the latter may not be restricted to plants as originally postulated, as PtcK clearly demonstrates that proteins similar to the PORR family exist outside of the plant lineage. 

Another PPR protein candidate, PtcJ is predicted to contain a meprin and TRAF-C homology (MATH) domain, a domain involved in peptide cleavage and processing, signal transduction, and ubiquitination [41]. However, given that these are unlikely functions for a PPR protein and that the similarity of PtcJ to the MATH domain is weak, PtcJ may exhibit a scenario similar to PtcK in that the MATH domain is not catalytic, but rather may be an RNA binding domain. 

Lastly, TPRpred analysis of PtcL provided a low probability of the candidate being a PPR protein (Table 1), despite the fact that there were at least six PPR motifs, and no other features were detected. It is, therefore, important to note that in previous work in *Trypanosoma brucei, *a PPR candidate (TbPPR9) had been identified with a TPRpred score even lower than that obtained for PtcL, but the *T. brucei *protein was later shown to be a bona fide PPR protein [19]. Considering this, and taking into account the degenerate nature of PPR motifs, it is not unreasonable to postulate that PtcL, despite its low probability score, may also be a bona fide PPR protein. 

### 3.2. A *D. discoideum *PPR Candidate Localizes to Mitochondria

**Figure 3:**
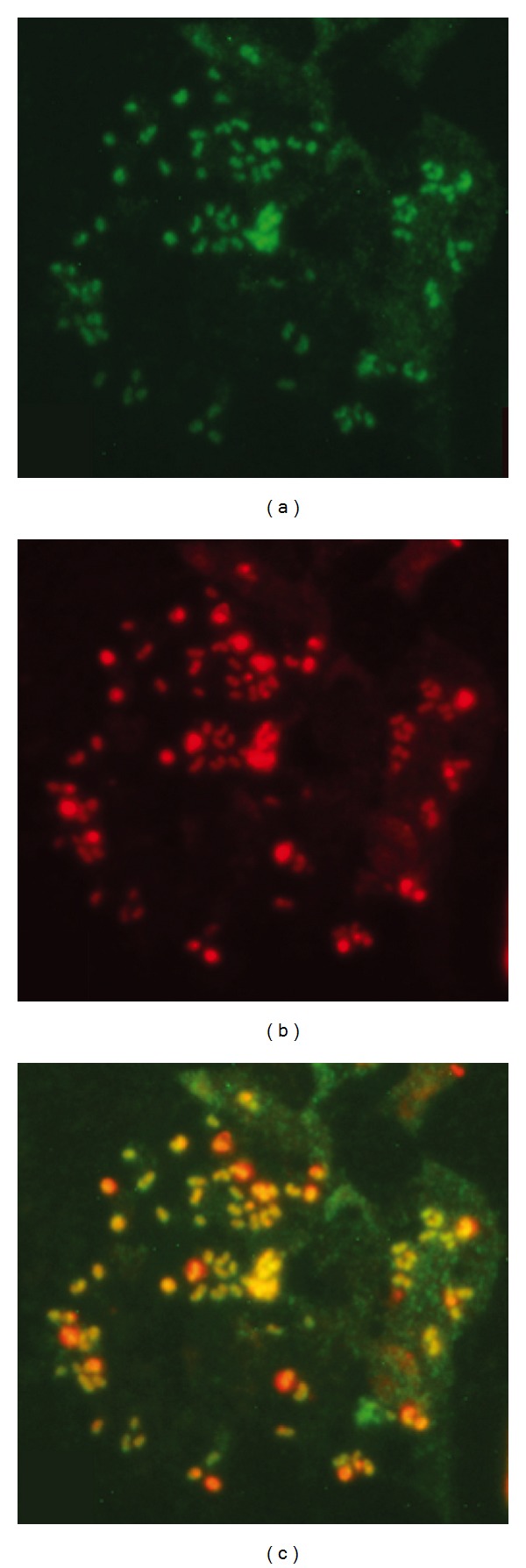
Subcellular localisation of PtcB. Fluorescence microscopy of *D. discoideum* cells (a) expressing a PtcB:GFP fusion protein,(b) stained with Mitotracker, (c) indicating that the fusion protein and the mitochondria colocalise.

Additional *in silico *analysis of the protein sequences indicated that most of these candidates are predicted to contain N-terminal mitochondrial targeting signals (Table 1) as inferred by the software program Mitoprot [42]. Following their initial identification, one PPR candidate, PtcB, was selected for further analysis. To confirm its mitochondrial localisation, a fusion gene was created containing the 5′ end of the *ptcB *gene (414 bp), encoding the putative mitochondrial targeting signal, fused to the gene encoding the green fluorescent protein (GFP). When this construct was transformed and expressed in *D. discoideum *cells, the PtcB:GFP fusion protein colocalised with the mitochondria (Figure 3), confirming that PtcB is indeed a mitochondrial protein and suggesting a physiological role for the protein within this organelle. 

### 3.3. Antisense Inhibition of *D. discoideum *PPR Expression Results in Slower Growth, a Phenotype Associated with Mitochondrial Dysfunction

**Figure 4:**
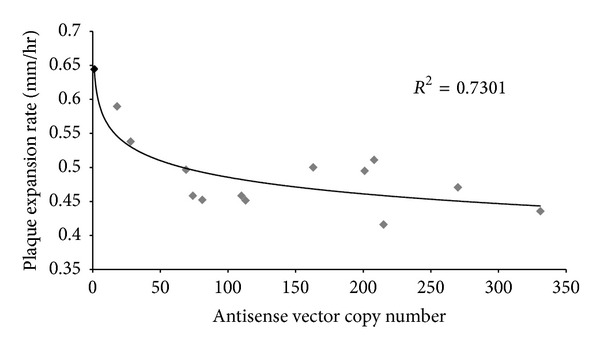
Plaque expansion rates of *ptcB *antisense transformants on *Escherichia coli *B2 lawns. Plaque expansion rates for *ptcB *antisense transformants are plotted against the copy number of the antisense construct present in each transformant, a reflection of the level of antisense inhibition. The number of copies of the antisense construct in each transformant was determined using qPCR. All transformants are shaded in grey while the wild type parental strain is in black.

To confirm a functional role of the* D. discoideum *PPR protein PtcB in the mitochondria, the expression of *ptcB* was knocked down via antisense inhibition. This involved cloning a portion of the *ptcB *gene (414 bp) into the *D. discoideum *expression vector pDNeo2 [43] in the antisense orientation relative to the actin 6 promoter. Expression of the *ptcB *gene fragment from this promoter will synthesise an antisense RNA transcript complementary to the endogenous *ptcB *mRNA sequence. Upon transformation of *D. discoideum *with vector DNA, the expression vector randomly integrates into the genome, whereby a single founding vector molecule will replicate at the integration site creating a random number of multimers [44]. As a result of this unique co-insertional replication mechanism, each *D. discoideum *transformant contains a different number of copies of the antisense construct and consequently, each transformant exhibits a different level of antisense inhibition [27]. This feature allows the antisense inhibition of a gene in a dosage-dependent manner. Following transformation of *D. discoideum *cells with the *ptcB *antisense construct, 13 antisense transformants were isolated. To establish whether PtcB has an essential role in mitochondrial function, the growth rates for these transformants were determined by growing the transformants on bacterial lawns. In *D. discoideum, *growth has been demonstrated to be one of the first phenotypes affected by non-functioning mitochondria, and thus slower growth serves as an indicator of mitochondrial dysfunction [27, 36]. This is because mitochondrial dysfunction triggers a cascade of pathways in *D. discoideum *that favour the repression of ATP consuming processes such as growth [27, 36]. Antisense inhibition of *ptcB* resulted in slower plaque expansion rates on bacterial lawns, and the severity of this phenotype correlated with the level of antisense inhibition of *ptcB *as indicated by the number of antisense constructs present in each of the transformants (Figure 4). The slower growth of *D. discoideum *antisense transformants confirms the important role PPR proteins play in *D. discoideum *mitochondrial function. Delayed growth upon knockdown of PPR-encoding genes has also been observed in trypanosomes [18, 19], and in plants, PPR mutants are known to display phenotypes associated with chloroplast or mitochondrial dysfunction, including cytoplasmic male sterility, negative effects on embryonic development, and defective photosynthetic ability [5, 45, 46].

### 3.4. *D. discoideum *PPR Proteins Possess Homologs in the Cellular Slime Mould Lineage

To gain further insight into the evolution of PPR proteins in the cellular slime mould lineage, we searched for PPR protein-encoding genes in the genomes of three other cellular slime moulds, *Dictyostelium purpureum, Dictyostelium fasciculatum, *and *Polysphondylium pallidum*. Interestingly, the search led to the identification of what seemed to be homologs of most of the PPR proteins previously identified in *D. discoideum *(Table 2). For most of these homologs it was confirmed by TPRpred analysis that they contain PPR motifs (Table 3). In two of the proteins, however, PPR motifs could not be detected (protein accession numbers XP_003284803 and XP_003286762), despite the fact that each of the candidates displayed a high level of homology to a specific *D. discoideum *PPR protein (Table 2). The failure to identify any PPR motifs within these proteins may be a result of weak conservation of their PPR motifs. 

None of the identified PPR proteins seem to have homologs in organisms outside of the cellular slime mould lineage (data not shown). A similar pattern of high conservation of PPR homologs has also been observed previously for non-plant PPR proteins in closely related species [17, 19]. The high level of conservation not only demonstrates the importance of PPR proteins in mitochondrial function, but also suggests a specific role for each of these homologs. It is therefore likely that these proteins fulfil more similar functions required by all four cellular slime mould species. However, some PPR homologs could only be found in *D. discoideum *and *D. purpureum,* indicating a potential conserved function of the proteins in these organisms, which is either not required or is performed by a different protein in *P. pallidum *and *D. fasciculatum *mitochondria. In addition, our sequence analysis also revealed that some of the cellular slime moulds possess PPR proteins which are not found in any of the others (Table 4). These candidates may represent unique PPR proteins that perform functions only required in these cellular slime moulds. However, it is noteworthy to mention that one of these proteins, XP_003291713 from *D. purpureum, *may have a putative homolog in *D. discoideum *(protein accession number XP_644522), but no PPR motifs were detected in the *D. discoideum* protein by TPRpred (data not shown).

## 4. Conclusions

The presence of PPR proteins in the model eukaryote *D. discoideum* suggests an important role for these proteins in the regulation of the mitochondrial transcriptome. This is supported by the antisense inhibition of one of the PPR-encoding genes, *ptcB,* yielding phenotypes characteristic of mitochondrial dysfunction in the protist. While the precise function of PPR proteins remains to be elucidated, it is clear that the function of most of these proteins is conserved supported by the presence of homologs in other cellular slime moulds. The potential functions of these proteins seem to differ from the function of RNA editing type PPR proteins in *N. gruberi* but may be analogous to the function of trypanosomal PPR proteins in modifying the stability of mitochondrial transcripts. One of the PPR candidates identified, PtcE, also contains a C-terminal methyltransferase domain, which has not been identified in any PPR protein to date, further attesting to the significance of studying PPR proteins in the *D. discoideum* model. The potential methyltransferase activity and the presence of other domains in some of the PPR proteins, therefore, suggest some unique functions for PPR proteins in *D. discoideum *mitochondria which have not been observed for PPR proteins of other organisms before. Thus, the functional study of PPR proteins in *D. discoideum *will provide an elegant system for investigating the important role PPR proteins played not only in protozoan mitochondrial gene expression but also more generally in non-plant organisms.

## Figures and Tables

**Table 1:** Bioinformatic analysis of putative *D. discoideum *pentatricopeptide repeat candidates. The probability of the helical repeats being pentatricopeptide repeats and the number of motifs were predicted using TPRpred, and the probability of mitochondrial targeting was predicted using Mitoprot.

Gene information			Protein information			
Gene	Chromosome location	Gene size (bp)	Length (amino acids)	Probability of PPR (%)	Number of PPR motifs	Probability of mitochondrial targeting (%)
*ptcA *	6	1,518	505	99.99	4	98
*ptcB *	5	1,783	528	100	9	91
*ptcC *	5	2,076	611	100	11	80
*ptcD *	5	4,321	1,405	97.18	7	23
*ptcE *	1	2,334	746	100	5	53
*ptcF *	3	1,596	531	100	6	93
*ptcG *	2	1,371	423	100	5	92
*ptcH *	3	3,247	1,057	96.37	4	88
*ptcI *	2	3,737	1,163	96.62	10	89
*ptcJ *	2	3,868	1,258	53.44	5	95
*ptcK *	2	3,623	1,148	100	11	54
*ptcL *	6	3,351	1,116	8.28	6	66

**Table 2:** Putative homologs of *D. discoideum *PPR proteins in other cellular slime moulds. The presence of a homolog is noted by the NCBI protein accession number while the absence of a clear homolog is denoted by “—”. Also, indicated in the parentheses are the levels of amino acid identity/similarity (%), respectively, for each protein compared to the *D. discoideum *homolog as determined by end to end pairwise alignments.

*D. discoideum* protein	*D. purpureum* homolog	*P. pallidum* homolog	*D. fasciculatum* homolog
PtcA	XP_003289503 (26.5/43.6)	—	—
PtcB	XP_003288427 (52.9/71.2)	EFA79424 (16/22.7)	EGG14329 (32.1/49.4)
PtcC	XP_003290170 (48.1/66.6)	EFA76720 (37.4/55.5)	EGG22645 (39.3/62.1)
PtcD	XP_003284803 (21.7/33.1)	—	—
PtcE	XP_003288663 (67.5/78.5)	EFA82229 (46.3/60.9)	EGG13534 (17.5/24.6)
PtcF	XP_003294037 (49.9/64.8)	EFA79525 (27.5/50.3)	EGG15096 (29/49)
PtcG	XP_003284179 (61.9/75.1)	EFA75260 (28.2/37)	EGG14213 (49.5/64.8)
PtcH	XP_003286839 (24.6/41.2)	—	—
PtcI	XP_003285976 (24.4/41.5)	—	—
PtcJ	XP_003291714 (25.6/44.5)	—	—
PtcK	XP_003293255 (27.2/44.3)	—	—
PtcL	XP_003286762 (25.8/42.2)	—	—

**Table 3:** Bioinformatic analysis of *D. discoideum *PPR protein homologs in other cellular slime moulds. The probability of helical repeats being PPR and the predicted number of motifs were determined using TPRpred.

Organism	NCBI protein accession number	PPR probability (%)	Number of PPR motifs
*D. purpureum *	XP_003289503	100	9
*D. purpureum *	XP_003288427	100	9
*D. purpureum *	XP_003290170	100	12
*D. purpureum *	XP_003284803	0	0
*D. purpureum *	XP_003288663	100	5
*D. purpureum *	XP_003294037	100	6
*D. purpureum *	XP_003284179	100	5
*D. purpureum *	XP_003286839	100	9
*D. purpureum *	XP_003285976	100	15
*D. purpureum *	XP_003291714	0.89	3
*D. purpureum *	XP_003293255	99.96	9
*D. purpureum *	XP_003286762	0	0
*P. pallidum *	EFA79424	100	9
*P. pallidum *	EFA76720	100	13
*P. pallidum *	EFA82229	100	3
*P. pallidum *	EFA79525	100	8
*P. pallidum *	EFA75260	100	5
*D. fasciculatum *	EGG14329	100	9
*D. fasciculatum *	EGG22645	100	12
*D. fasciculatum *	EGG13534	97.20	3
*D. fasciculatum *	EGG15096	100	8
*D. fasciculatum *	EGG14213	100	6

**Table 4:** Bioinformatic analysis of unique PPR proteins found in one, but not in other cellular slime moulds. The probability of helical repeats being PPR and the predicted number of motifs were determined using TPRpred.

Organism	NCBI protein accession number	PPR probability (%)	Number of PPR motifs
*D. purpureum *	XP_003291713	99.55	8
*P. pallidum *	EFA82227	64.34	3
*P. pallidum *	EFA76758	51.88	6
*P. pallidum *	EFA80531	100	15
*D. fasciculatum *	EGG19875	99.98	8
*D. fasciculatum *	EGG23890	100	12

## Abbreviations

PPR: Pentatricopeptide repeat
PtcA-L: Pentatricopeptide repeat-containing protein A-L
PORR: Plant organelle RNA recognition.
